# Are Inventory Based and Remotely Sensed Above-Ground Biomass Estimates Consistent?

**DOI:** 10.1371/journal.pone.0074170

**Published:** 2013-09-19

**Authors:** Timothy C. Hill, Mathew Williams, A. Anthony Bloom, Edward T. A. Mitchard, Casey M. Ryan

**Affiliations:** 1 Department of Earth and Environmental Science, University of St Andrews, St Andrews, United Kingdom; 2 School of GeoScience, The University of Edinburgh, Edinburgh, United Kingdom; DOE Pacific Northwest National Laboratory, United States of America

## Abstract

Carbon emissions resulting from deforestation and forest degradation are poorly known at local, national and global scales. In part, this lack of knowledge results from uncertain above-ground biomass estimates. It is generally assumed that using more sophisticated methods of estimating above-ground biomass, which make use of remote sensing, will improve accuracy. We examine this assumption by calculating, and then comparing, above-ground biomass area density (AGBD) estimates from studies with differing levels of methodological sophistication. We consider estimates based on information from nine different studies at the scale of Africa, Mozambique and a 1160 km^2^ study area within Mozambique. The true AGBD is not known for these scales and so accuracy cannot be determined. Instead we consider the overall precision of estimates by grouping different studies. Since an the accuracy of an estimate cannot exceed its precision, this approach provides an upper limit on the overall accuracy of the group. This reveals poor precision at all scales, even between studies that are based on conceptually similar approaches. Mean AGBD estimates for Africa vary from 19.9 to 44.3 Mg ha^−1^, for Mozambique from 12.7 to 68.3 Mg ha^−1^, and for the 1160 km^2^ study area estimates range from 35.6 to 102.4 Mg ha^−1^. The original uncertainty estimates for each study, when available, are generally small in comparison with the differences between mean biomass estimates of different studies. We find that increasing methodological sophistication does not appear to result in improved precision of AGBD estimates, and moreover, inadequate estimates of uncertainty obscure any improvements in accuracy. Therefore, despite the clear advantages of remote sensing, there is a need to improve remotely sensed AGBD estimates if they are to provide accurate information on above-ground biomass. In particular, more robust and comprehensive uncertainty estimates are needed.

## Introduction

There is international recognition that greenhouse gas emissions from land-use change (LUC) are significant and need to be reduced as part of a wider strategy to mitigate climate change. Initiatives such as Reducing Emissions from Deforestation and forest Degradation and enhancing carbon stocks through sustainable forest management (REDD+) are designed to provide the financial incentives to reduce these emissions in developing countries [1]. The net flux of carbon (C) from tropical land has recently been estimated to be a source of 1.3±0.7 Pg C year^−1^ [2]. Tropical emissions of 2.9±0.5 Pg C year^–1^ resulting from deforestation are only partially offset by uptake from tropical forest regrowth of 1.6±0.5 Pg C year^−1^ [2]. Other estimates of the emissions from tropical LUC differ both in their magnitude (0.89 to 1.52 Pg C year^−1^) and uncertainty (±0.20 to ±0.31 Pg C year^−1^) [3]. A recent estimate [4] estimates gross carbon emissions from tropical regions of 0.81 Pg C year^–1^ (with a 90% prediction interval of between 0.57 and 1.22 Pg C year^–1^), values which are only 25 to 50% of the gross emissions used by Pan, et al., (2011). Estimates of C fluxes from LUC are therefore poorly constrained at global [5] and regional scales [3]. Without accurate estimates of LUC fluxes, policy in this area risks misdirecting resources, crediting emission reductions that have already occurred, or failing to incentivise reductions in countries with high (but currently unquantified) LUC fluxes. To avoid these pitfalls, schemes such as REDD+ require robust estimates of LUC and associated fluxes to develop reference levels and provide sufficient confidence in monitoring to enable investment [6].

LUC fluxes can be estimated through a range of different approaches, including mapping changes in the spatial extent of land-cover classes, each with an estimated mean AGB, and ideally uncertainty [3]. In order to attain accurate LUC flux estimates with this method, the mean above-ground biomass (AGB) must be accurately known, as must changes in AGB through time. At the sub-national scales, on which REDD+ type policies are currently being implemented [7], [8], a range of approaches for estimating AGB are available. The most generic approaches rely on “default” values of above-ground biomass area density (AGBD) for individual land-cover classes which are often specific to continents, countries or biomes (note that we distinguish the area-integrated AGB of a region, country or continent from the mean per hectare above-ground biomass area density which we refer to as AGBD). These generic approaches then rely on simple extrapolation between time points to estimate change in AGB. A potential alternative to these approaches comes from the spatially explicit estimates of AGBD using readily available moderate-resolution remotely sensed observations of canopy height and reflectance [9]. These moderate-resolution remote sensing approaches enable maps of the natural variability of AGBD across a landscape to be estimated with the potential for large scale remotely sensed change estimates.

The United Nations Framework Convention on Climate Change (UNFCCC) define a hierarchy of “Tiers” to differentiate between the various methods of C accounting that rely on different levels of sophistication in estimating AGBD and the areal extent and emissions from land use and land use change activities [9], [10]. Tier 1 is based on regional or global estimates of AGBD (i.e. the IPCC default values) and an assumption that changes in stocks equate to emissions; Tier 2 builds on Tier 1 with the inclusion of country specific data on AGBD for dominant land uses and emission estimates from other activities; and Tier 3 provides high resolution information at sub-national scales and may include modelling of emissions based on observed changes in stocks. For example, new satellite-based approaches have been described as being appropriate for the UNFCCC’s Tier 3 reporting requirements [11], although it should be noted that the actual Tier level of a given approach to estimating AGBD inherently depends on the specifics of any subsequent analysis. Implicitly there is an expectation that transitioning up the Tiers will provide increased accuracy [9], [10], [12]. In this study we assess this expectation by comparing estimates of AGBD that are suitable for Tier 1, 2 and 3 at the scale of: (i) Africa, (ii) Mozambique, and (iii) a small study area of 1160 km^2^ within Mozambique. The small study area is chosen due to the availability of high resolution (i.e. 25 m by 25 m) remote sensing estimates of AGBD which have been created by fusing radar data with a network of forest plots.

The concepts of uncertainty and error, precision and accuracy are important to this study. Definitions of both uncertainty and error vary, and in some cases are they are treated as being synonymous [13]. Here we use the terms to express different concepts [14]. An error is the difference between a measurement and the true value, such that subtracting the error from the measurement would theoretically result in the true value. Errors can be random or systematic. Uncertainty represents the distribution of differences between the true value and a range of estimates and is normally given at a particular confidence level.

The precision, or repeatability, of a measurement is the closeness of agreement between measured values and therefore depends only on the distribution of random errors. Precision does not consider systematic errors or, indeed, the true value. Accuracy is the agreement between the measurement and the true value, and, depends on both the random and systematic errors. The accuracy of a measurement has to be lower than its precision, except in the special case with no systematic error, when they are equal.

In this study we apply these concepts to biomass estimation, estimating the overall precision of an ensemble of estimates from nine different sources (Table 1). It is important to note that any individual estimate could be of higher accuracy and/or precision than the ensemble precision. Despite the true AGBD being highly uncertain (i.e. unknown) at continental scales, we are able to test the expectation that an ensemble of accurate, and therefore precise, estimates have to be in close agreement with one another. Furthermore a robust estimate should be associated with an uncertainty estimate that adequately describes the overall uncertainty. In the case of multiple robust estimates, the individual uncertainty estimates should explain the distribution of AGBD estimates.

**Table 1:** Dataset summary.

Source	Scales (Appropriate Tier)	Overview of base data.	Uncertainties Considered
FRA 1990 [18]	Africa (Tier 1)	Based on country scale values. Natural forest cover 568,000,000 ha in 1980 and 527,600,000 ha in 1990. AGBD for forested areas of 133 Mg ha^−1^. Plantation coverage is 0.5%, but plantation AGBD not available. Therefore plantations were not included. *F* = 5.3%.	None.
FRA 1990 [18]	Mozambique (Tier 1)	Based on country scale values. Natural forest cover 17,329,000 ha in 1990 with an annual deforestation rate of 0.7%. AGBD for forested areas of 80 Mg ha^−1^. Plantation coverage is 0.2%, but plantation AGBD not available. Therefore plantations were not included.	None.
Brown and Gaston 1995 [43]	Mozambique (Tier 2)	AGBD for woody formations from a Geographic Information System (GIS) model with a 5 km by 5 km resolution, driven by the FAO data describing climate, soils, population and vegetation distribution. AGBD estimate for woody formations in Mozambique was 57 Mg ha^−1^ in ∼1980. Converted to AGBD for Mozambique using the FAO’s 1980 Mozambique’s total forest cover area estimate of 17,505,400 ha.	None.
FRA 2000 [21]	Africa (Tier 1)	Based on country scale values. Forest cover including plantations was 649,866,000 ha in 2000 with an annual deforestation rate of 0.8%. AGBD for forested areas of 109 Mg ha^−1^. No adjustment factor applied, *F* = 0%.	None.
FRA 2000 Remote sensing [21]	Africa (Tier 3)	Based on Landsat products. Forest area including plantations was 519,000,000 (±37,000,000) ha (standard error of the mean). The annual deforestation rate was 0.34% (±0.06%) year^−1^ (standard error of the mean). Uses the ‘f3’ definition of forests which “*is the broadest and includes the classes of long fallow and a higher fraction (one-third) of the fragmented forest class than the f2 definition*” [21]. The average AGBD of forests was 109 Mg ha^−1^. *F* = 4.5%.	Incomplete remote sensing coverage: Random sampling only includes 10% of area considered.
FRA 2000 [21]	Mozambique (Tier 2)	Based on country scale values. Forest cover including plantations was 30,601,000 ha in 2000, with an annual deforestation rate of 0.2%. AGBD for forested areas of 55 Mg ha^−1^.	None.
FRA 2005 [22]	Africa (Tier 1)	Based on country scale values. Africa’s forest cover area, including plantations, was 699,361,000 ha in 1990, 655,613,000 ha in 2000, and 635,412,000 ha in 2005. Between 1990 and 2000, the annual deforestation rate was 0.64%, and between 2000 and 2005 the deforestation rate was 0.62%. No AGBD for forests was presented in the 2005 FRA report and so a value of 109 Mg ha^−1^ was used from the earlier FRA 2000 report. *F = *0%.	None.
Drigo et al., 2008 [44]	Mozambique (Tier 3)	Based on sub-country values and MODIS products and with a 2.5 by 2.5 km resolution. Mozambique’s total AGB for woody stock was 1,615,091,000 Mg in 2004.	None.
FRA 2010 [20]	Africa (Tier 1)	Based on country scale values. Africa’s forest cover area, including plantations, was 749,238,000 ha in 1990, 708,564,000 ha in 2000, and 691,468,000 ha in 2005, and 674,419,000 ha in 2010. Combined above-ground and below-ground area density was 172.7 Mg ha^−1^ in 1990, 174.87 Mg ha^−1^ in 2000, 175.4 Mg ha^−1^ in 2005, and 176.0 Mg ha^−1^ in 2010. The root-shoot ratio for all years was 0.24. No Adjustment factor needed as this is the reference estimate.	None.
FRA 2010 [20]	Mozambique (Tier 2)	Based on country scale values. Mozambique’s forest cover area, including plantations, was 43,378,000 ha in 1990, 41,188,000 ha in 2000, 40,079,000 ha in 2005, and 39,022,000 ha in 2010. The carbon density of forests in Mozambique was 43 MgC ha^−1^. The carbon fraction was 0.47.	None.
Saatchi et al., 2011 [19]	Africa (Tier 3)	Based on GLAS, MODIS, QSCAT, and SRTM products. Estimates have a 1 by 1 km resolution. The mean total above-ground carbon in biomass for forests with 10% tree cover was 47,902,000,000 MgC. The carbon fraction was 0.5. *F = *1.2%.	At 95% confidence, a low estimate of total above-ground carbon in biomass 44,584,000,000 MgC and high estimate of 51,616,000,000 MgC were generated using bootstrapping cross-validation. Uncertainty estimate includes observation, sampling and prediction errors. Uncertainty is scaled assuming pixels to be spatially uncorrelated.
Saatchi et al., 2011 [19]	Mozambique (Tier 3)	Based on GLAS, MODIS, QSCAT, and SRTM products. Estimates have a 1 by 1 km resolution. The mean total above-ground carbon in biomass for forests with 10% tree cover was 1,714,000,000 MgC. The carbon fraction was 0.5.	At 95% confidence, a low estimate of total above-ground carbon in biomass 1,655,000,000 MgC and high estimate of 1,714,000,000 MgC were generated using bootstrapping cross-validation. Uncertainty estimate includes observation, sampling and prediction errors. Uncertainty is scaled assuming pixels to be spatially uncorrelated.
Saatchi et al., 2011 [19]	Study Area (Tier 3)	Based on GLAS, MODIS, QSCAT, and SRTM products. Estimates have a 1 by 1 km resolution. The carbon fraction was 0.5.	We use the larger pixel (100 ha) 95% confidence interval uncertainty of ±53%. Under the assumption of independent random errors [19], we calculated the study area relative uncertainty to be ±1.56%.
Ryan et al. 2012 [16]	Study Area (Tier 3)	Based on ALOS-PALSAR with a 25 by 25 m resolution. Total carbon stored in AGB was 2,130,000 MgC in 2007 and 1,980,000 MgC in 2010. A carbon fraction of 0.48 was used [15].	Regression uncertainty estimates generated by using a boot strapping approach.
Baccini et al., 2012 [11]	Africa (Tier 3)	Based on GLAS and MODIS products with a 500 by 500 m resolution. The total above-ground carbon in biomass for vegetation in tropical Africa 64,500,000,000 MgC. The carbon fraction was 0.5. *F* = 2.0%.	The uncertainty of ±8,600,000,000 MgC represents the 95% confidence interval. GLAS regression errors and modelling errors. Uncertainty is scaled assuming a complete correlation below a scale of 500 km and no correlation above this scale.
Baccini et al., 2012 [11]	Mozambique (Tier 3)	Based on GLAS and MODIS products with a 500 by 500 m resolution. The total above-ground carbon in biomass for vegetation in tropical Africa 2,687,000,000 MgC. The area of Mozambique was clipped to the “tropical region”. As the extent of the clipped area was not provided we use a land area of 78,638,000 ha [20]. The carbon fraction was 0.5.	The uncertainty range minimum was 2,676,000,000 MgC,with a maximum of 2,695,000,000 MgC.
Baccini et al., 2012 [11]	Study Area (Tier 3)	Based on GLAS and MODIS products with a 500 by 500 m resolution. AGBD was determined from the 463 m by 463 m pixel dat. The carbon fraction was 0.5.	Uncertainty estimates were not available at this scale.

A summary of the datasets used in this study, further details are included in the supporting information.

In this study we convert existing estimates of AGB into comparable AGBD estimates and then calculate the overall ensemble precision to provide insights into the overall uncertainty of current AGBD estimates and their robustness.

## Methods

### 3.1 Overview of Methods

Based on a search of peer reviewed literature and the United Nations Food and Agriculture Organization’s (FAO) Forest Resource Assessment (FRA) we calculated estimates of AGBD for Africa, Mozambique and a smaller 1160 km^2^ region in central Mozambique (Table 1 and File S1). Estimates of total AGB were converted into mean AGBD to facilitate comparisons between the three scales. The 1160 km^2^ study area is within Gorongosa and Nhamatanda Districts [15], [16]. The area is dominated by Miombo woodland and has a seasonal wet-dry climate. Since the end of the Mozambican civil war in 1992, the area has undergone rapid land use change catalysed by the resettlement of rural areas and the rebuilding of road infrastructure. Losses of forest carbon within the area are mainly driven by small holder agriculture and charcoal production.

### 3.2 Choice of Data Sources

We aimed to include as many independent estimates as possible (Table 1 and File S1). Data sources that simply restate earlier data (normally from the FAO FRA reports) were not included in the comparison. However, multiple FRA report estimates were included to capture the variability in FRA estimates [17]. The key features of each estimate are summarised in Table 1 and described in detail in the supplementary material.

### 3.3 Definitions of Above-ground Biomass, Forest and Deforestation

We use the FAO definition of forest: non-agricultural ecosystems with a minimum of 10% crown cover of trees [18]. Where data from remote sensing estimates was defined by crown cover, we pick the same 10% definition [19]. However some remote sensing estimates consider all sources of above-ground biomass [16], and so are expected to exhibit slightly higher AGBD than estimates using the FRA definition. FRA reports consider deforestation to be land-use changes that reduce the crown cover of trees to less than the 10% threshold. Reductions in crown cover that do not cross this threshold (i.e. from closed canopy to open canopy forest) are defined as degradation [18]. Degradation is not considered in mono-temporal FRA reports, but is implicitly included in multi-temporal remote sensing estimates of AGBD.

### 3.4 Adjusting African Estimates to the same Selection of Countries

Many of the studies include a different selection of countries in their definition of Africa based on latitude, low proportion of forests or small size [11], [18], [19]. If uncorrected for, this would lead to the country selection impacting estimates of AGBD for Africa. These differing African areas needed to be normalised before comparisons can be made. The FRA 2010 estimate spans the most complete selection of countries and was used as the reference [20]. To rescale the other AGBD estimates, we calculated adjustment factors to scale AGBD estimates as if they covered the same countries as the FRA 2010 report. These factors adjust for the AGB excluded from each estimate. Factors were calculated separately for each estimate. The adjustment factor, *F* was calculated as
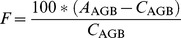
where *A*_AGB_ is the total FRA 2010 AGB of all countries in the FRA 2010 Africa estimate, and *C*_AGB_ is the total FRA 2010 AGB of the countries considered by a particular estimate. The original AGB estimates for Africa were then increased by the following adjustment factors: 5.3% (FRA 1990), 0.0% (FRA 2000), 0.0% (FRA 2005), 1.2% (Saatchi, et al., 2011) and 2.0% (Baccini, et al., 2012). The use of these adjustment factor means that all AGBD estimates for Africa are comparable to the FRA 2010 selection of countries.

The FRA 2000 remote sensing dataset [21] represented an incomplete, sub-sampled survey area, however it was not clear which countries were included and which were not. Therefore an alternative approach to calculating the adjustment factor was taken. The estimate of forest area, based on country data, corresponding to the region surveyed by the remote sensing was 95.7% of the total FRA 2010 Africa forest area [21]. Therefore, under the assumption that missing locations were random, an adjustment factor of 4.5% was applied to the remote sensing estimate.

### 3.5 Calculation of AGBD

AGBD estimates are presented as a mean above-ground biomass area density, in units of Mg of dry mass per hectare (Mg ha^−1^). The basic calculation for this area density is: the total AGB (Mg) for the region (including any adjustment factor) divided by the total land area of the region (ha). Where sources provide estimates in terms of carbon we use a carbon fraction between 0.47 and 0.5 (depending on the study) to convert to the dry mass (Table 1).

### 3.6 Conversion of Uncertainties

Individual uncertainty estimates have been included in our comparison when available from the literature. Uncertainty estimates are presented as 95% confidence intervals, assuming a normal distribution. We note that the exact assumptions and scope of each uncertainty estimate differ (Table 1).

### 3.7 Calculation of AGBD Ranges

Ensemble uncertainty estimates and precisions are not calculated due to the small number of estimates in each grouping. Instead, ranges of AGBD are calculated as the difference between maximum and minimum estimates for a particular grouping. These ranges exceed the 95% confidence interval uncertainties. The groupings considered were either scale specific (Africa, Mozambique or the study area), Tier specific (Tier 1, 2 or 3), or a combination of the two.

## Results

**Figure 1:**
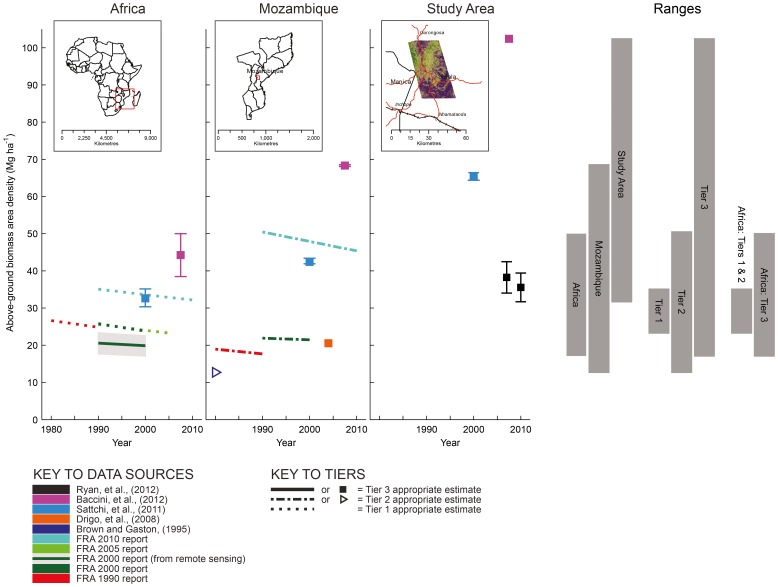
Estimated above-ground biomass area density (AGBD) at the scale of Africa, Mozambique and the study area. Colours are used to denote the primary source of information. Depending on temporal extent, the style of line or marker is used indicate if an estimate is Tier 1, 2 or 3 appropriate. Where available, uncertainties have been scaled to 95% confidence levels and are indicated with error bars or shading (in the case of the FRA 2000 report). To the right of the plots, bars are used to indicate the ranges of three groupings (i.e. different scales, different Tiers, or Tiers 1 and 2 versus Tier 3 for Africa).

The collected inventory and remote sensing estimates of AGBD span three decades (Figure 1). The minimum estimate of AGBD for Africa, excluding uncertainties, was 19.9 Mg ha^−1^ and the maximum was 44.3 Mg ha^−1^ (Table 2). For Mozambique the minimum estimate, excluding uncertainties, was 12.7 Mg ha^−1^ and the maximum was 68.3 Mg ha^−1^. Finally, the minimum estimate for the study area, also excluding uncertainties, was 35.6 Mg ha^−1^ and the maximum was 102.4 Mg ha^−1^.

**Table 2:** Above-ground biomass area density.

Source	Africa, Mg ha^−1^(±95% CI)	Mozambique, Mg ha^−1^(±95% CI)	Study Area, Mg ha^−1^(±95% CI)	Tier(s)
FRA 1990 [18]	24.9 → 26.6	17.7 → 19.0		1 & 2
Brown and Gaston 1995 [43]		12.7		2
FRA 2000 [21]	23.8 → 25.8	21.5 → 21.9		1 & 2
FRA 2000 Remote sensing [21]	19.9 (±2.8)→ 20.6 (±2.8)			3
FRA 2005 [22]	23.3 →25.6			1
Drigo et al., 2008 [44]		20.5		3
FRA 2010 [20]	32.2 →35.1	45.4 →50.5		1 & 2
Saatchi et al., 2011 [19]	32.6 (−2.3, +2.5)	42.4 (−0.5, +1.0)	65.4 (−1.0, +0.0)	3
Ryan et al. 2012 [16]			35.6 (±3.9)→ 38.3 (±4.2)	3
Baccini et al., 2012 [11]	44.3 (±5.8)	68.3 (−0.3, +0.2)	102.4	3

The main source of the estimate is indicated in the first column. Where estimates from multiple time points exist an arrow is used to indicate lower and upper values. Where available, uncertainties corresponding to the 95% confidence intervals are shown in brackets. The highest of UNFCCC’s Tiers for which the estimate is appropriate, is indicated in the final column.

The range of AGBD estimates at the study area-scale exceeded the range of estimates for larger areas (e.g. Africa and Mozambique, Figure 1). AGBD estimates specifically for the study area – which are all Tier 3 appropriate – have a range of 70.7 Mg ha^−1^, exceeding the range of Tier 1, 2 and 3 appropriate estimates of AGBD derived at the scale of Africa (range of 32.9 Mg ha^−1^) and Mozambique (range of 55.8 Mg ha^−1^). Considering just African scale AGBD estimates, Tier 3 appropriate approaches have a greater range than the Tiers 1 and 2 appropriate methods (a range of 32.9 Mg ha^−1^ versus a range of 11.8 Mg ha^−1^). The range of estimates is indicative of the overall level of precision of the different approaches.

## Discussion

**Figure 2:**
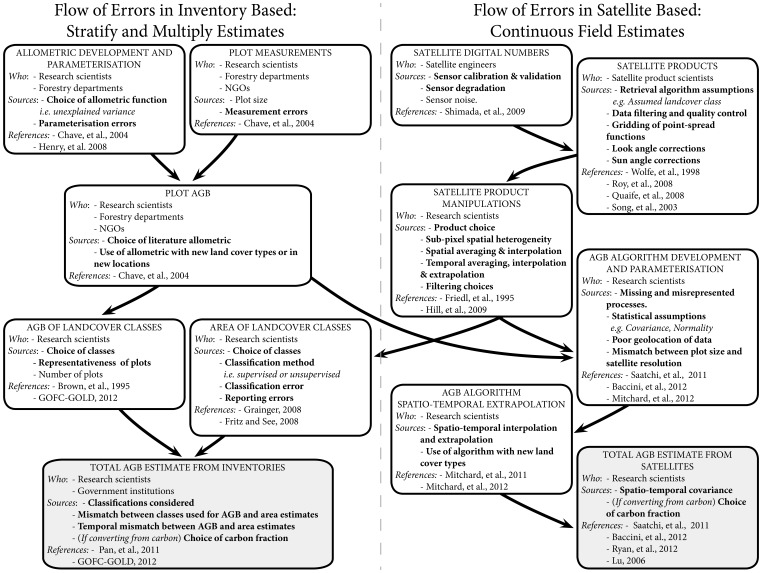
Flow of errors in inventory and satellite based AGB estimates. Boxes are used to highlight particular steps that contribute to the overall uncertainty. The groups of users that typically carry out each step, and specific sources of error are indicated in the text within each box. Where an error is likely to be systematic, the descriptive text is shown in bold. Arrows indicate the flow of information and therefore errors. This diagram is for illustrative purposes and should not be seen as an attempt to set out a comprehensive list of all errors, for all estimates of AGB. The references included are: [17], [19], [23], [28], [32], [33], [34], [35], [36], [37], [38], [39], [40], [41], [42].

Our study reveals that as a result of low overall ensemble precision, there is a lack of consistency between estimates at all spatial scales and methodological sophistication (Figure 1). Our analysis does not indicate if all the estimates are inaccurate, but it appears at least that the majority of estimates need improvement in their estimates of magnitude and/or uncertainty of AGBD. Tier 3 appropriate estimates of AGBD tend to rely more heavily on satellite products than estimates appropriate to the lower Tiers (Table 1). The lower precision of Tier 3 AGBD estimates compared to Tiers 1 and 2 should not be interpreted as evidence that satellite methods are less accurate. This lowering of precision with increasing methodological sophistication could actually hide an increase in accuracy, if the Tier 1 and 2 appropriate methods were sufficiently inaccurate. That is to say the Tier 1 and 2 appropriate methods could have high precision (i.e. good agreement), but low accuracy (i.e. poor agreement with the truth). Indeed the majority of Tier 1 and 2 appropriate estimates all depend on similar inventory data [20], [21], [22] and allometrics (Figure 2) and so errors are likely to have significant systematic components.

Previous studies have documented the lack of robustness of inventory estimates [3], [17]. Indeed, Tier 1 appropriate AGB estimates have been revised by successive FRA reports. However, without uncertainties, the significance of the revisions to FRA estimates is difficult to ascertain, i.e. are the updates large with respect to a claimed uncertainty? The lack of independent Tier 1 and 2 appropriate estimates limits our ability to identify inconsistent estimates, and consequently our ability to test for improvements with Tier 3 appropriate methods. Until independent validation can be carried out, the lack of agreement between Tier 3 appropriate methods and the limited number of independent Tier 1 and Tier 2 appropriate estimates has potential to bias policies relying on a single source of AGB data.

Uncertainty estimates are not available for many of the current AGBD estimates, and when available, the majority of these uncertainty estimates are likely to be too small. At the scales of Africa, Mozambique, or the study area, only two independent pairs of the AGBD uncertainty estimates overlap at 95% confidence levels (e.g. the “*FRA 2010: Africa” with “Saatchi: Africa”,* and the “*FRA 2000: Africa remote sensing” with “FRA 2005: Africa”,* Figure 1). The number of errors contributing to uncertainty in ABGD estimates from both inventory and satellite based approaches is numerous (Figure 2). Sources of uncertainty in inventory based estimates of AGBD include measurement and reporting errors, errors resulting from too few and/or poorly placed plots, and poorly known allometry [23]. By providing spatially continuous observations, remote sensing can be used to extrapolate information from field observations. Implicitly, however, estimates based on remote sensing rely on field observations for parameterisation and corroboration, and are therefore also subject to the same uncertainties that impact plot estimates of AGB. Additional uncertainty in remotely sensed estimates comes from errors on the satellite digital numbers, the generation of satellite products, spatio-temporal mismatches between data sources, and other statistical and structural errors in the modelled relationship between satellite observations and actual AGB. Many of these errors result in systematic errors that will not cancel with spatio-temporal averaging. Because of these factors the precise scope of AGBD uncertainty estimates is often unclear with only a subset of errors actually being explicitly included (Figure 2).

As there are no currently agreed upon ‘true’ values for AGBD [24], the actual accuracy of an AGBD estimate cannot be calculated. Precision does not relate to the true value and is easier to estimate. However, because many of the errors involved in estimating AGBD from satellite observations are systematic (Figure 2), the precision of an estimate might be expected to be significantly better than the accuracy. Despite this, there is notably low precision and poor consistency between Tier 3 estimates based on the same core data source (e.g. the satellite-borne LiDAR measurements [11], [19]). Furthermore the nature of the sources of error, some of which are spatially and temporally correlated, means that it is important, though extremely difficult, to robustly estimate uncertainty over a range of spatiotemporal scales. For example, Saatchi, et al. (2011) estimate uncertainties at the finest spatial (i.e. pixel) scale to be between ±6% and ±53%. When scaling these uncertainties to national or regional scales, however, error correlation between neighbouring locations is not accounted for, leading to unrealistically small relative uncertainty at regional and national scales [19] (Figure 1).

Given that uncertainty comprises spatially uncorrelated errors, which cancel with spatial averaging, and correlated errors, which do not cancel, it is perhaps surprising that the agreement between Tier 3 AGBD estimates is only slightly better at national and continental scales than at the small scale of the study area (Figure 1). Miombo woodland is also one of the more challenging land-cover types to map and monitor for biomass change [25] and (relative to the larger spatial scales) an even greater disagreement at these fine scales might be expected.

Different definitions of which land cover to include in general classification of forests and how to do so (e.g. inclusion/exclusion of plantations, different classifications of forest types) will introduce systematic biases in AGB estimates (Figure 2). These biases are evident in all Tier levels and complicate inter-comparisons. In some cases the direction of the bias, if not the magnitude, can be deduced, excluding regions with less than 10% canopy cover will clearly reduce the total AGB [19]. However, in others cases decisions made about the classes to use are tied to a particular method and will result in a bias that is largely unknown.

A recent FAO forestry paper published a new approach to estimating forest cover loss [26]. This estimate only considers forest cover and was not included in this study. However comparisons to the earlier FAO remote sensing estimate for the period 1990 to 2000 reveal a close agreement for forest area, but much lower agreement for change in forest area [26]. For Africa, the earlier report predicts a rate of forest area loss approximately twice that of the more recent report. This highlights the additional uncertainties that might be expected when these AGBD estimates are used to detect deforestation.

Despite the current poor precision and unknown accuracy of Tier 3 appropriate AGBD estimates, there are clear advantages of recent remote sensing approaches over national inventories, with significant potential for further improvement. Currently, only the most recent FRA reports provide a Tier 1 appropriate estimate accounting for the change in AGBD [20]. Earlier FRA reports used a fixed carbon density per land cover class and were only capable of detecting changes in biomass resulting from land-cover change [18], [21], [22]. High resolution AGBD estimates from recent remote sensing approaches are not limited in this regard, as they do not rely on land-cover classifications (Figure 2). Furthermore, Tier 3 appropriate AGBD have the potential to be repeatable (though not all are currently multi-temporal), methodologically consistent, spatially continuous and applicable over a range of scales [11], [19], [27]. It is also important to note that, theoretically, remote sensing AGBD estimates could be independently verified with plot inventories; though such comparisons are only truly valid if the plots are excluded from the development of the remote sensing estimate, cover all landcover types and the full biomass range, and are spatially independent from any plot data used for calibration [28]. This approach would require significant resources, but would provide a means of estimating the actual uncertainties on Tier 3 appropriate AGBD estimates.

## Conclusions

There are many calls to improve the accuracy of AGB, deforestation and forest degradation estimates [23], [29], [30]. This study shows that there is no clear improvement in precision when using more sophisticated approaches based on satellite data. However, precision is much easier to characterise than accuracy, and whilst it can provide an idea of the best-case accuracy, it does not account for systematic errors which are a potentially large source of uncertainty. We would therefore add to the call for improved accuracy the need to improve the reporting of uncertainty on these estimates. The basis of current AGB uncertainty is often poorly described and can be of unknown origin or elicited from expert opinion, e.g. [12], [31]. The scope of uncertainties can be unknown, or limited to a subset of the possible error sources, (e.g. [20], [21], [22]). Moreover, these uncertainty estimates are rarely independently tested. Our analysis shows the majority of (and potentially all) current AGBD estimates are overly confident in their level of uncertainty. We recommend that wherever possible all high resolution estimates of AGBD should be accompanied by independently corroborated uncertainties over the full range of aggregate scales that AGBD estimates are provided on, from the finest spatial resolution to global scales. In order to provide the independent corroboration of AGB uncertainty estimates, we recommend collating a network of ground comparison sites with a common measurement protocol that are of sufficient spatial extent to be comparable with moderate-resolution satellite estimates. These sites should be explicitly excluded from the development, parameterisation and testing of individual estimates.

## Supporting Information

File S1 **Descriptions of the information used to convert each estimate to AGBD.**
(DOC)

## References

[1] Stickler CM, Coe MT, Costa MH, Nepstad DC, McGrath DG, et al.. (2013) Dependence of hydropower energy generation on forests in the Amazon Basin at local and regional scales. Proceedings of the National Academy of Sciences. 10.1073/pnas.1215331110 PMC3677497 23671098

[2] Pan Y, Birdsey RA, Fang J, Houghton R, Kauppi PE, et al (2011) A large and persistent carbon sink in the world’s forests. Science 333: 988–993. 21764754 10.1126/science.1201609

[3] Malhi Y (2010) The carbon balance of tropical forest regions, 1990–2005. Current Opinion in Environmental Sustainability 2: 237–244.

[4] Harris NL, Brown S, Hagen SC, Saatchi SS, Petrova S, et al (2012) Baseline Map of Carbon Emissions from Deforestation in Tropical Regions. Science 336: 1573–1576. 22723420 10.1126/science.1217962

[5] Houghton RA (2010) How well do we know the flux of CO(2) from land-use change? Tellus Series B-Chemical and Physical Meteorology 62: 337–351.

[6] Venter O, Watson JEM, Meijaard E, Laurance WF, Possingham HP (2010) Avoiding Unintended Outcomes from REDD. Conservation Biology 24: 5–6. 20121835 10.1111/j.1523-1739.2009.01391.x

[7] Blom B, Sunderland T, Murdiyarso D (2010) Getting REDD to work locally: lessons learned from integrated conservation and development projects. Environmental Science & Policy 13: 164–172.

[8] Streck C, Gomez-Echeverri L, Gutman P, Loisel C, Werksman J (2009) REDD+ Institutional Options Assessment. Developing an Efficient, Effective, and Equitable Institutional Framework for REDD+ under the UNFCCC. Meridian Institute.

[9] Herold M, Roman-Cuesta RM, Mollicone D, Hirata Y, Van Laake P, et al (2011) Options for monitoring and estimating historical carbon emissions from forest degradation in the context of REDD+. Carbon Balance Manag 6: 13. 22115360 10.1186/1750-0680-6-13 PMC3233497

[10] Penman J (2006) Good practice guidance for land use, land-use change and forestry. Hayama, Kanagawa, Japan: Published by the Institute for Global Environmental Strategies for the IPCC.

[11] Baccini A, Goetz SJ, Walker WS, Laporte NT, Sun M, et al (2012) Estimated carbon dioxide emissions from tropical deforestation improved by carbon-density maps. Nature Climate Change 2: 182–185.

[12] Angelsen A, Brown S, Loisel C, Peskett L, Streck C, et al.. (2009) Reducing Emissions from Deforestation and Forest Degradation (REDD): An Options Assessment Report. Meridian Institute.

[13] Taylor JR (1997) An introduction to error analysis : the study of uncertainties in physical measurements. Sausalito, Calif.: University Science.

[14] BIPM IEC, IFCC ILAC, IUPAC, etal. (2012) The international vocabulary of metrology–basic and general concepts and associated terms (VIM): JCGM.

[15] Ryan CM, Williams M, Grace J (2011) Above- and Belowground Carbon Stocks in a Miombo Woodland Landscape of Mozambique. Biotropica 43: 423–432.

[16] Ryan CM, Hill TC, Woollen E, Ghee C, Mitchard E, et al (2012) Quantifying small-scale deforestation and forest degradation in African woodlands using radar imagery. Global Change Biology 18: 243–257.

[17] Grainger A (2008) Difficulties in tracking the long-term global trend in tropical forest area. Proceedings of the National Academy of Sciences of the United States of America 105: 818–823. 18184819 10.1073/pnas.0703015105 PMC2206620

[18] FAO (1993) Forest resources assessment 1990: Tropical countries. Rome: FAO.

[19] Saatchi SS, Harris NL, Brown S, Lefsky M, Mitchard ET, et al (2011) Benchmark map of forest carbon stocks in tropical regions across three continents. Proc Natl Acad Sci U S A 108: 9899–9904. 21628575 10.1073/pnas.1019576108 PMC3116381

[20] FAO (2010) Global forest resources assessment 2010: Main report. Rome: Food and Agriculture Organization of the United Nations. xxxi, 340 p.

[21] FAO (2001) Global forest resources assessment 2000: Main report. Rome; [Great Britain]: Food and Agriculture Organization of the United Nations. xxvii, 479 p. p.

[22] FAO (2006) Global forest resources assessment 2005: Progress towards sustainable forest management. Rome: Food and Agriculture Organization of the United Nations. xxvii, 320 p. p.

[23] Chave J, Condit R, Aguilar S, Hernandez A, Lao S, et al (2004) Error propagation and scaling for tropical forest biomass estimates. Philosophical Transactions of the Royal Society of London Series B-Biological Sciences 359: 409–420. 10.1098/rstb.2003.1425 PMC1693335 15212093

[24] Clark DB, Clark DA (2000) Landscape-scale variation in forest structure and biomass in a tropical rain forest. Forest Ecology and Management 137: 185–198.

[25] Grainger A (1999) Constraints on modelling the deforestation and degradation of tropical open woodlands. Global Ecology and Biogeography 8: 179–190.

[26] Lindquist EJ, D’Annunzio R, Gerrand A, MacDicken K, Achard F, et al.. (2012) Global forest land-use change 1990–2005, FAO & JRC.

[27] Le Toan T, Quegan S, Davidson MWJ, Balzter H, Paillou P, et al (2011) The BIOMASS mission: Mapping global forest biomass to better understand the terrestrial carbon cycle. Remote Sensing of Environment 115: 2850–2860.

[28] Mitchard ETA, Saatchi SS, Lewis SL, Feldpausch TR, Gerard FF, et al (2011) Comment on ‘A first map of tropical Africa’s above-ground biomass derived from satellite imagery’. Environmental Research Letters 6: 049001.

[29] Baker DJ, Richards G, Grainger A, Gonzalez P, Brown S, et al (2010) Achieving forest carbon information with higher certainty: A five-part plan. Environmental Science & Policy 13: 249–260.

[30] Achard F, Stibig HJ, Eva H, Lindquist E, Bouvet A, et al (2010) Estimating tropical deforestation from Earth observation data. Carbon Management 1: 271–287.

[31] Achard F, Eva HD, Mayaux P, Stibig HJ, Belward A (2004) Improved estimates of net carbon emissions from land cover change in the tropics for the 1990s. Global Biogeochemical Cycles 18.

[32] Shimada M, Isoguchi O, Tadono T, Isono K (2009) PALSAR Radiometric and Geometric Calibration. Ieee Transactions on Geoscience and Remote Sensing 47: 3915–3932.

[33] Wolfe RE, Roy DP, Vermote E (1998) MODIS land data storage, gridding, and compositing methodology: Level 2 grid. Ieee Transactions on Geoscience and Remote Sensing 36: 1324–1338.

[34] Roy DP, Ju J, Lewis P, Schaaf C, Gao F, et al (2008) Multi-temporal MODIS-Landsat data fusion for relative radiometric normalization, gap filling, and prediction of Landsat data. Remote Sensing of Environment 112: 3112–3130.

[35] Quaife T, Lewis P, De Kauwe M, Williams M, Law BE, et al (2008) Assimilating canopy reflectance data into an ecosystem model with an Ensemble Kalman Filter. Remote Sensing of Environment 112: 1347–1364.

[36] Song CH, Woodcock CE (2003) Monitoring forest succession with multitemporal Landsat images: Factors of uncertainty. Ieee Transactions on Geoscience and Remote Sensing 41: 2557–2567.

[37] Henry M, Picard N, Trotta C, Manlay RJ, Valentini R, et al (2011) Estimating Tree Biomass of Sub-Saharan African Forests: a Review of Available Allometric Equations. Silva Fennica 45: 477–569.

[38] Fritz S, See L (2008) Identifying and quantifying uncertainty and spatial disagreement in the comparison of Global Land Cover for different applications. Global Change Biology 14: 1057–1075.

[39] GOFC-GOLD (2012) A sourcebook of methods and procedures for monitoring and reporting anthropogenic greenhouse gas emissions and removals associated with deforestation, gains and losses of carbon stocks in forests remaining forests, and forestation.: GOFC-GOLD Land Cover Project Office, Wageningen University, The Netherlands.

[40] Lu DS (2006) The potential and challenge of remote sensing-based biomass estimation. International Journal of Remote Sensing 27: 1297–1328.

[41] Mitchard ETA, Saatchi SS, White LJT, Abernethy KA, Jeffery KJ, et al (2012) Mapping tropical forest biomass with radar and spaceborne LiDAR in Lope National Park, Gabon: overcoming problems of high biomass and persistent cloud. Biogeosciences 9: 179–191.

[42] Brown IF, Martinelli LA, Thomas WW, Moreira MZ, Ferreira CAC, et al (1995) Uncertainty in the Biomass of Amazonian Forests - an Example from Rondonia, Brazil. Forest Ecology and Management 75: 175–189.

[43] Brown S, Gaston G (1995) Use of forest inventories and geographic information systems to estimate biomass density of tropical forests: Application to tropical Africa. Environmental Monitoring and Assessment 38: 157–168. 24197942 10.1007/BF00546760

[44] Drigo R, Cuambe C, Lorenzini M, Marzoli A, Macuacua J, et al.. (2008) WISDOM Mozambique: Wood energy supply/demand analysis applying the WISDOM methodology. Maputo: Ministério de Agricultura, Direcção Nacional de Terras e Florestas (DNTF). 67 p.

