# Supplementary material for: Are Inventory Based and Remotely Sensed Above-Ground Biomass Estimates Consistent?
Source: PLoS One. 2013 Sep 19;8(9):e74170. doi: 10.1371/journal.pone.0074170 (PMC3777937; doi:10.1371/journal.pone.0074170)
Supplement: File S1 — Descriptions of the information used to convert each estimate to AGBD. (DOC) [file pone.0074170.s001.doc]

# S1: Supporting Information

## Details on individual estimates

*Estimate 1: FRA 1990 report: Africa.*

Africa’s natural forest cover area was 568,000,000 ha in 1980 and 527,600,000 ha in 1990 (Table 4 in ). Plantations covered 3,000,000 ha, 0.5% of the natural forest cover, but their mean AGBD was not available, and so plantations were not included in the AGBD estimate. The effect of excluding plantations is expected to be small. The annual deforestation rate was 0.7% year^-1^ (Table 4a in ). The average AGBD for forested areas was 133 Mg ha^-1^ (Table 3a in ). Due to several countries not being considered in the estimate, AGBD was adjusted by 5.3% to provide a comparable estimate to the FRA 2010 Africa report. This estimate is Tier 1 appropriate.

*Estimate 2: FRA 1990 report: Mozambique*

Mozambique’s natural forest cover area was 17,329,000 ha in 1990, the annual deforestation rate was 0.7 % (Table 4a in ). Plantations cover 40,000 ha, 0.2% of the natural forest cover, but mean AGBD was not available and they were not included. Mozambique’s total land area was 78,409,000 ha (Table 4a in ). The AGBD of forested regions was 80 Mg ha^-1^ (Table 3a in ). This estimate is Tier 2 appropriate.

*Estimate 3: Brown and Gaston (1995): Mozambique*

Brown and Gaston (1995) used a Geographic Information System (GIS) based model on a 5 km by 5 km resolution, which was driven by the Food and Agriculture Organisation's (FAO) data describing climate, soils, population and vegetation distribution. The AGBD estimate for woody formations in Mozambique was 57 Mg ha^-1^ in ~1980. We normalise this estimate using the FAO’s 1980 Mozambique’s total forest cover area estimate (17,505,400 ha) and the total land area of Mozambique (78,409,000 ha) (Table 4a in ). We classify the estimate as being Tier 2 appropriate, as Brown and Gaston (1995) caution against using sub-country level estimates from this approach due to the coarse resolution of the driving data used in the GIS model .

*Estimate 4, FRA 2000 report: Africa*

Africa’s forest cover area, including plantations (see appendix 2 in ), was 649,866,000 ha in 2000 (Table 17-1 in ). The annual deforestation rate is 0.8% year^-1^ (Table 11-1 in ). The total land area of Africa was stated to be 2,978,000,000 ha, (Table 1-2 in ). The AGBD of forests was 109 Mg ha^-1^ (Table 1-2 in ). No adjustment factor was applied. This estimate is Tier 1 appropriate.

*Estimate 5, FRA 2000 report: Africa (remote sensing)*

Africa’s forest cover area assessed from remote sensing, including plantations (appendix 2 in ), was 519,000,000 ha in 2000, with a standard error of the mean of 37,000,000 ha (Table 1-3 in ). The 2000 remote sensing forest cover estimate uses the ‘f3’ definition of forests which “*is the broadest and includes the classes of long fallow and a higher fraction (one-third) of the fragmented forest class than the f2 definition*”, (Chapter 46 in ). The annual deforestation rate was 0.34 % (± 0.06%) year^-1^ (standard error of the mean) (Table 1-4 in ). Standard errors were multiplied by 1.96 to convert them to a 95% confidence interval. The reported total land area of Africa was 2,978,000,000 ha, (Table 1-2 in ). The average AGBD of forests was 109 Mg ha^-1^, (Table 1-2 in ). Due to incomplete coverage of Africa, biomass area density was adjusted by 4.5% to provide a comparable estimate to the FRA 2010 Africa report. This estimate is Tier 3 appropriate.

*Estimate 6, FAO 2000 report: Mozambique*

Mozambique’s forest cover area, including plantations, was 30,601,000 ha in 2000 (Table 17-1 in ). The annual deforestation rate was 0.2% year^-1^ (Table 17-1 in ). The reported total land area of Mozambique was 78,409,000 ha. Forests had an AGBD of 55 Mg ha^-1^ (Table 17-1 in ). This estimate is Tier 2 appropriate.

*Estimate 7, FRA 2005 report: Africa*

Africa’s forest cover area, including plantations, was 699,361,000 ha in 1990, 655,613,000 ha in 2000, and 635,412,000 ha in 2005 (Table 4 in ). Between 1990 and 2000, the annual deforestation rate was 0.64 % year^-1^, and between 2000 and 2005 the deforestation rate was 0.62 % year^-1^ (Table 4 in ). The reported total land area of Africa was 2,974,000,011 ha, (Table 1 in ). No AGBD for forests was presented in the 2005 FRA report and so a value of 109 Mg ha^-1^ was used from the earlier FRA 2000 report (Table 1-2 in ). No adjustment factor was applied. This estimate is Tier 1 appropriate.

*Estimate 8: Drigo et al. 2008 Wisdom report: Mozambique*

Mozambique's total AGB for woody stock was 1,615,091,000 Mg in 2004 (Table 5 in ). The reported land area of Mozambique was 78,638,000 ha (Table 1 in ). This estimate is Tier 3 appropriate.

*Estimate 9, FRA 2010 report: Africa*

Africa’s forest cover area, including plantations, was 749,238,000 ha in 1990, 708,564,000 ha in 2000, and 691,468,000 ha in 2005, and 674,419,000 ha in 2010 (Tables 2.1 and 2.7 in ). Biomass was presented in the report as a combined above-ground and below-ground density, which was 172.7 Mg ha^-1^ in 1990, 174.87 Mg ha^-1^ in 2000, 175.4 Mg ha^-1^ in 2005, and 176.0 Mg ha^-1^ in 2010 (Table 2.19 in ). The root-shoot ratio for all years was 0.24, (Table 2.18 in ). The reported total land area of Africa was 2,974,000,011 ha (Table 1 in ). This estimate is Tier 1 appropriate.

*Estimate 10, FAO 2010 report: Mozambique.*

Mozambique’s forest cover area, including plantations, was 43,378,000 ha in 1990, 41,188,000 ha in 2000, 40,079,000 ha in 2005, and 39,022,000 ha in 2010 (Table 3 in ). The carbon density of forests in Mozambique was 43 MgC ha^-1^ (Table 11 in ). A carbon fraction of 0.47 was assumed . The root-shoot ratio for Eastern and Southern Africa (0.26) was used to convert to AGBD (Table 2.18 in ). The reported land area of Mozambique was 78,638,000 ha (Table 1 in ). This estimate is Tier 2 appropriate.

*Estimate 11, Saatchi, et al., (2011): Africa.*

The mean total above-ground carbon in biomass for forests with 10% tree cover was 47,902,000,000 MgC . A low estimate of 44,584,000,000 MgC and high estimate of 51,616,000,000 MgC were also provided (Table S3a in ). High and low values represent the 95% confidence interval derived from bootstrapping cross-validation. The total land area of Africa used for this calculation was 2,974,000,011 ha, (Table 1 in ). A carbon fraction of 0.5 was used . Due to several countries not being considered in the estimate, the AGBD estimate was adjusted by 1.2% to provide a comparable estimate to the FRA 2010 Africa report. This estimate is Tier 3 appropriate.

*Estimate 12, Saatchi, et al., (2011): Mozambique.*

The mean total above-ground carbon in biomass for forests with 10% tree cover was 1,714,000,000 MgC . A low estimate of 1,655,000,000 MgC and high estimate of 1,714,000,000 MgC were also provided (Table S3a in ). High and low values represent the 95% confidence interval derived from bootstrapping cross-validation. The reported area of Mozambique was 79,000,000 ha (Table S3a in ). A carbon fraction of 0.5 was used . This estimate is Tier 3 appropriate.

*Estimate 13, Saatchi, et al., (2011): Study Area.*

The mean above-ground carbon in biomass for the study area was determined by averaging all 1 km x 1 km AGB pixel estimates within the study area . We use the larger pixel (100 ha) 95% confidence interval uncertainty of ±53 %. Under the assumption of independent random errors , we calculated the study area relative uncertainty to be ± 1.56% . Though we do not use it, we note that the smaller pixel level uncertainty of ±6 % would equate to a study area relative uncertainty of ±0.18 %. This estimate is Tier 3 appropriate.

*Estimate 14, Ryan, et al., (2012): Study area.*

The study area was 116,000 ha and the total carbon stored in AGB was 2,130,000 ± 120,000 MgC in 2007 and 1,980,000 ± 110,000 MgC in 2010. The errors shown indicate one standard deviation and were converted to 95% confidence intervals by multiplying by 1.96. A carbon fraction of 0.48 was assumed . This estimate is Tier 3 appropriate.

*Estimate 15, Baccini, et al., (2012): Africa.*

The total above-ground carbon in biomass for vegetation in tropical Africa 64,500,000,000 ± 8,600,000,000 MgC . The error represents the 95% confidence interval. Not all African countries were considered in the estimate. The total land area of Africa used for this calculation was 2,974,000,011 ha (Table 1 in ). A carbon fraction of 0.5 was used. Due to several countries not being considered in the estimate, AGBD was adjusted by 2.0% to provide a comparable estimate to the FRA 2010 Africa report. This estimate is Tier 3 appropriate.

*Estimate 16, Baccini, et al., (2012): Mozambique.*

The above-ground carbon in biomass for vegetation in Mozambique was 2,687,000,000 MgC . A range on this estimate is given with a minimum of 2,676,000,000 MgC, and a maximum of 2,695,000,000 MgC . The area of Mozambique was clipped to the “tropical region”. As the extent of the clipped area was not provided we use a land area of 78,638,000 ha (Table 1 in ). Thus this estimate should be an underestimate of AGBD. A carbon fraction of 0.5 was used. This estimate is Tier 3 appropriate.

*Estimate 17, Baccini, et al., (2012): Study Area.*

AGBD was determined from the 463 m by 463 m pixel data . The average AGBD of all pixels within the study area used to calculate the mean AGBD [5]. This estimate is Tier 3 appropriate.

## References

45. FAO (1993) Forest resources assessment 1990: Tropical countries. Rome: FAO.

46. Brown S, Gaston G (1995) Use of forest inventories and geographic information systems to estimate biomass density of tropical forests: Application to tropical Africa. Environmental Monitoring and Assessment 38: 157-168.

47. FAO (2001) Global forest resources assessment 2000: Main report. Rome ; [Great Britain]: Food and Agriculture Organization of the United Nations. xxvii, 479 p. p.

48. FAO (2006) Global forest resources assessment 2005: Progress towards sustainable forest management. Rome: Food and Agriculture Organization of the United Nations. xxvii, 320 p. p.

49. FAO (2010) Global forest resources assessment 2010: Main report. Rome: Food and Agriculture Organization of the United Nations. xxxi, 340 p. p.

50. Drigo R, Cuambe C, Lorenzini M, Marzoli A, Macuacua J, et al. (2008) WISDOM Mozambique: Wood energy supply/demand analysis applying the WISDOM methodology. Maputo: Ministério de Agricultura, Direcção Nacional de Terras e Florestas (DNTF). 67 p.

51. Saatchi SS, Harris NL, Brown S, Lefsky M, Mitchard ET, et al. (2011) Benchmark map of forest carbon stocks in tropical regions across three continents. Proc Natl Acad Sci U S A 108: 9899-9904.

52. Ryan CM, Hill TC, Woollen E, Ghee C, Mitchard E, et al. (2012) Quantifying small-scale deforestation and forest degradation in African woodlands using radar imagery. Global Change Biology 18: 243-257.

53. Ryan CM, Williams M, Grace J (2011) Above- and Belowground Carbon Stocks in a Miombo Woodland Landscape of Mozambique. Biotropica 43: 423-432.

54. Baccini A, Goetz SJ, Walker WS, Laporte NT, Sun M, et al. (2012) Estimated carbon dioxide emissions from tropical deforestation improved by carbon-density maps. Nature Climate Change 2: 182-185.
